# Erianin inhibits the progression of DDP-resistant lung adenocarcinoma by regulating the Wnt/β-catenin pathway and activating the caspase-3 for apoptosis in vitro and in vivo

**DOI:** 10.1186/s41065-024-00351-x

**Published:** 2024-11-27

**Authors:** Lingxue Tang, Yiling Ruan, Beibei Wang, Mingjun Zhang, Jie Xue, Tong Wang

**Affiliations:** 1 Department of General practice, The Second Affiliated Hospital of Anhui Medical University, Hefei, Anhui China; 2 Department of Oncology, The Second Affiliated Hospital of Anhui Medical University, Hefei, Anhui China; 3 Department of General Practice, Suixi County Hospital, Huaibei, Anhui China

**Keywords:** Erianin, DDP-resistance, Lung adenocarcinoma, Apoptosis, Progression

## Abstract

**Background:**

Platinum-based chemotherapy is one of the main treatments for lung adenocarcinoma (LUAD). However, the toxic side effects and drug resistance of chemotherapeutic drugs on normal cells are still a thorny problem in clinical treatment. Dendrobium is one of the three largest genera of Orchidaceous family, which has ornamental and medicinal value. Dendrobium is mainly distributed in the tropics and subtropics of South Asia, Oceania and other regions, with 1547 species of Dendrobium currently known. In China, “Shi hu” and “Tie pi shi hu” are well-known traditional medicines and have been included in the Chinese Pharmacopoeia (Editorial Board of Chinese Pharmacopoeia, 2020). Erianin is a natural product isolated from Dendrobium and is considered as a potential anticancer molecule due to its remarkable anti-tumor effects through various mechanisms, among which induced cancer cell apoptosis, inhibited invasion and migration. This study preliminarily explored the mechanism of Erianin inhibiting the progression of cisplatin (DDP) resistant LUAD in vivo and in vitro.

**Methods:**

The effect of Erianin on the proliferation of DDP-resistant LUAD cells was detected by CCK-8 assay, wound healing assay and cloning assay. Transwell assay was used to evaluate the effect of Erianin on cell invasion and migration. The changes of cell cycle and apoptosis were detected by flow cytometry and TUNEL assay. Finally, the effects of Erianin on cell function and signaling pathway-related protein expression in vivo and in vitro were examined based on the enrichment analysis.

**Results:**

Erianin could inhibit the proliferation, invasion and migration, induce apoptosis, altered cell cycle of DDP-resistant LUAD cells, and reverse the resistance to DDP. Western blotting results showed that Erianin exerted its anti-tumor effects by regulating the Wnt/β-catenin cascade in DDP-resistant LUAD cells.

**Conclusion:**

Erianin may exerted its anti-tumor effect in DDP-resistant LUAD cells by regulating the Wnt3/β-Catenin/Survivin/Bcl-2/Caspase-3/Cyclin D1 axis.

## Introduction

Lung cancer is one of the deadliest malignancies, with the highest incidence and mortality in the world [1]. Platinum-based chemotherapy is the most common treatment for LUAD. Unfortunately, acquired drug resistance has become a serious obstacle [2]. Although previous studies have reported the mechanism of DDP-resistance, including increased resistance to cell apoptosis, abnormal expression of transporters, and enhanced DNA repair, the mechanism of resistance remains unknown in 20–30% of cases [3]. Reversing drug resistance in LUAD and improving the therapeutic effect of anti-tumor has become a hot topic of research in recent years. Traditional Chinese medicine (TCM) is considered as one of the adjuvant treatments in the standard treatment of LUAD and deserves further attention. The most basic theory of TCM in the treatment of LUAD is to enhance the anti-cancer immunity of the body and directly inhibit the growth, proliferation, invasion and migration of tumor cells when the body’s immunity is too weak and the tumor growth ability is too strong [4]. TCM has a variety of treatment methods, among which Chinese herbal medicine is the most important part [5]. In addition, TCM treatment after standard treatment can alleviate cancer-related symptoms and improve quality of life [6, 7]. It can also prolong the progression-free survival of stage II ~ IIIA patients, and reduce the mortality rate and the recurrence and metastasis rate after tumor surgery [8–10]. The combination of Chinese medicine and western medicine also can improve the sensitivity of chemotherapy and radiotherapy, reduce the toxicity and adverse reactions in the treatment of non-small cell lung cancer [11, 12].

In clinical treatment, Dendrobium extract has a significant therapeutic effect on improving the symptoms of lung cancer patients, and researchers have found that it has a significant anti lung cancer effect on nude mouse tumor models [13]. However, its specific substances and mechanisms of action in lung cancer treatment have not been comprehensively analyzed. The identification and analysis of TCM components are crucial and serve as the basis for elucidating the clinical efficacy of Chinese medicine. Network pharmacology has been widely used in the prediction of bioactive components and the mechanism study of TCM by revealing the multi-aspect relationship between the targets and components of TCM from the perspective of whole and system [14, 15]. The research of Zhao et al. screened the main potential active ingredients for the treatment of lung cancer through various methods such as network pharmacology, among which dibenzyl accounted for the highest proportion, including Eranin [16].

Erianin, a naturally sourced, highly effective and low toxic small molecule compound, is considered a potential cancer treatment, and it’s mainly derived from Dendrobium. In China, there are 74 species and 2 varieties of Dendrobium. In TCM, “Shi hu” and “Tie pi shi hu” are precious Chinese medicine, which is extracted from the fresh or dried stems of various Dendrobium plants. It has the effects of nourishing Yin and clearing heat, promoting fluid and nourishing stomach, moistening lungs and relieving cough, etc. It has been used as a Chinese herbal medicine for thousands of years [17], and has been included in the Editorial Board of Chinese Pharmacopoeia (2020) [18]. Erianin is considered to be a representative indicator for the quality control of Dendrobium [19, 20]. In recent years, more and more studies have reported that Erianin has good anti-cancer activity in vivo and in vitro through blocking tumor cell cycle, inducing apoptosis, activating autophagy, inhibiting cell migration, and other ways [21–23]. For instance, Erianin can exert anti-tumor effects through the ERK pathway, ROS mediated JNK/c-Jun and AKT/mTOR pathways, PI3K/AKT/mTOR pathway, calcium/calmodulin dependent ferroptosis pathway [23–26]. In colon cancer, Erianin reversed the oxaliplatin-resistance by inhibiting the expression of STAT3 and P-gp [22], and exerted its anti-tumor angiogenesis effect [27]. In oral squamous cell cells, Erianin down-regulated the expression of PPT1, inhibited the growth of cancer cells, and induced cell apoptosis [28]. In triple-negative breast cancer, Erianin can induce apoptosis of cancer cells by activating the PI3K/Akt pathway [25, 29]. Study reported the effect of Erianin on the viability of lung cancer cell lines, finding that it induces cell death and G2/M phase arrest, and exerts its anti-cancer effect by inducing calcium/calmodulin dependent ferroptosis and inhibiting lung cancer cell migration [26]. The study results of Zhang et al. showed that Erianin could induce apoptosis of lung cancer cells and reduce cell viability, but had no obvious toxic effect on normal cells. It is speculated that the anti-lung cancer mechanism of Erianin is related to the inhibition of PI3K/Akt/mTOR pathway [25]. However, the mechanism of action of Erianin in chemotherapy-resistant lung cancer remains unclear.

There are few reports on the effect of Erianin on tumor chemotherapy sensitivity. In the study of Lv et al., they found that Erianin can affect the sensitivity of lung cancer cells to chemotherapy drug 5-Fu in vitro, showing concentration dependence, confirmed that Erianin promoted the inhibitory effect of 5-Fu on the growth of lung cancer transplant tumors through in vivo experiments. In addition, they also found that the combination therapy of Erianin and 5-Fu can improve the toxic effects of 5-Fu [30]. DDP-resistance is very common in cancers [31]. Research has confirmed that abnormal activation of β-catenin is involved in DDP-resistance in ovarian cancer [32], while inactivation of Wnt/β-catenin pathway can inhibit DDP-resistance in prostate cancer [33]. Abnormal activation of MAPK/ERK also involved in DDP-resistance in gastric and colorectal cancer [34, 35]. However, the role and mechanism of Erianin in DDP-resistance in LUAD need further exploration. In this study, the antitumor mechanism of Erianin in DDP-resistant LUAD was investigated. The aim is to provide more options and theoretical support for the clinical treatment of DDP-resistant LUAD patients.

## Materials and methods

### Cell culture and drug preparation

The human A549/DDP cell line was purchased from Procell Life Science&Technology Co., Ltd (Wuhan, China). We selected cells with good condition and cultured them using the complete medium (Ham’s F-12 K + 1 ~ 2 µg/mL DDP + 10% fetal bovine serum + 1% penicillin/streptomycin) at 37℃ with 5% CO_2_. According to the instructions, we added Erianin powder (MedChemExpress, Shanghai, China) into DMSO to prepare the stock solution, and stored it at -80℃. Then, we added Erianin solution into the fresh medium to prepare culture solution with different concentration based on the IC_50_ value. The control group was added DMSO in the same volume as the Erianin solution.

### Cell counting kit-8 (CCK-8) assay

We inoculated cells into 96-well plate at a density of 2*10^3^/well and cultured for 12 h until fully adherent. The culture medium of the control group and Erianin group were added into 96-well plates (100 µL/ well), and continued to incubate under standard conditions for 24 h. Subsequently, we replaced the medium with fresh medium containing 10µL CCK-8 solution (Biosharp, Shanghai, China), and continued to culture in the dark for 1 h. Then, we measured the absorbance of each well at a wavelength of 450 nm using an enzyme-linked immunosorbent assay. Cell viability (%) = (drug group absorbance - blank group absorbance) / (control group absorbance - blank group absorbance) *100%.

### Wound healing assay

Firstly, we marked the bottom surface of the 6-well plate to confirm that the scratch positions recorded during different time periods are consistent. Secondly, we inoculated the resuspended cells into the 6-well plate (5*10^5^/well), and used a 200 µl pipette to scratch vertically in the plate well when cells grew to 100% density. After washing with phosphate buffer saline (PBS), we starved and cultured cells with serum-free medium in control group and Erianin group for 24 h. Finally, we photographed the scratch areas for 0 and 24 h with a microscope, in order to analyze the scratch healing degree of the same site at different time periods.

### Colony formation assay

We inoculated the resuspended cells into the 6-well plate (1*10^3^/well), replaced the fresh medium in control group and Erianin group. Changed the culture medium every 2 ~ 3 days to ensure the concentration of Erianin. When cloned cell aggregate were observed under the microscope, the culture could be stopped, generally for about 2 weeks. After the cells were washed with PBS, we used the 4% paraformaldehyde fixing solution fixed the cells for 20 min, and stained with 0.1% crystal violet for 10 min. Finally, rinsed the cells with pure water, and record the results used the microscope after air dried.

### Transwell assay

The cell invasion and migration were by Transwell assay. We diluted the matrigel (Sigma, MO, USA) with serum-free medium in a ratio of 1:3, added it to the upper chamber of the chamber for 60 µL/well (Corning, MA, USA), and air-dry at 37℃ until gel formation (migration assay do not require Matrigel). Then, we prepared the cell suspension (invasion assay: 1*10^5^/well; migration assay: 2*10^4^/well) using serum-free medium and seeded it in the upper chamber (200 µL/well). Then, we added the complete culture medium in the control group and Erianin group into the lower chamber of the 24-well plate (600 µL/well). After incubation for 24 h, the cells penetrating the matrix were fixed in 4% paraformaldehyde for 20 min and stained with 0.1% crystal violet solution for 10 min. And we recorded the results use the inverted microscope.

### Flow cytometry assay

After 24 h of cell culture, we digested and centrifuged the cells from the control group and Erianin group, and collect them in tubes (3 ~ 7*10^6^/tube), respectively. Then, we add 1 mL pre-cooled PBS resuspension cells to each tube, centrifuged it again, and discard the supernatant. Subsequently, we added 1 mL of pre-cooled 70% ethanol, blew and mixed well, and placed it at 4ºC overnight. After that, we fixed the centrifuged cells immediately, discarded the supernatant, and washed the cells with 1mL pre-cooled PBS. Finally, we prepared the staining solution according to the instructions of the Cell Cycle and Apoptosis Analysis Kit (Beyotime, Shanghai, China), which the staining agent was propidium staining. Then, we added the staining solution into each sample and stain at 37 °C in the dark for 30 min. The fluorescence and light scattering were detected by flow cytometry after filtration the samples with a 70 μm cell screen.

### In vivo experiment

First, we collected the cells with good condition and re-suspended them with PBS, and injected into the right axilla of male Balb/c nude mice at 6-week-old (100 µL, about 2*10^6^ cells/mouse). Then, we measured the tumor length (L) and width (W) every 3 days. The volume (V) = LW^2^/2. After 10 days, the nude mice were randomly divided into control group and experimental group. The experimental group was intraperitoneally injected 100 mg/kg [26] Erianin once a day (Erianin powder was dissolved with DMSO and Corn Oil), while the control group was injected with the same dose of DMSO and Corn Oil. Both groups were injected continuously for 15 days. After stopping the injection for three days, euthanize the mice, dissect the subcutaneous tumors, and store them at -80℃ for future use.

### TUNEL assay

We soaked the tumor tissue and fixed it in 4% paraformaldehyde for 48 h, and fully dehydrated in an automatic dehydrator. After the tissue was embedded in paraffin, we cut it into thin slices with a microtome and fixed it on a slide. Then, we baked the slices in a constant temperature oven at 60℃ for 1 h and set aside for later use. The slices were dewaxed with xylene, (100%, 90%, 80%, 70%) ethanol aqueous solution in turn. After washing the slices with PBS, we balanced, labeled and fluorescently stained the samples according to the instructions of the One-step TUNEL In Situ Apoptosis Kit (Elabscience, Wuhan, China). Finally, the samples were sealed with anti-fluorescence quenched seal and observed by a standing fluorescence microscope in a dark environment and photographed.

### Western blotting analysis

We collected the protein lysates of samples with a mixture of RIPA Buffer + protease inhibitor + phosphatase inhibitor (Beyotime, Shanghai, China), and used the BCA reagent kit (Beyotime, Shanghai, China) to determine the protein concentration of each sample. Then, we separated the Stained Protein Ladder and an equal amount of protein samples (20 ~ 40 µg) from each group through sodium dodecyl sulfate polyacrylamide gel (SDS-PAGE), and transferred it to the polyvinylidene fluoride (PVDF) membranes by electricity. Subsequently, we placed the membranes in the Protein Free Rapid Blocking Buffer (Epizyme, Shanghai, China) for 20 min and incubated with primary antibodies at 4 °C overnight. On the second day, we washed the membranes with Tris-buffer saline (containing 0.1% Tween-20) for 3 times, and then incubate there with secondary antibody IgG-HRP at room temperature for 1.5 h. Finally, ECL reagent kit (Biosharp, Shanghai, China) was used for imaging.

The antibodies preparation for western blotting were as follows: β-Catenin (Affinity, 1:1000), p-β-Catenin (Affinity, 1:1000), Wnt3 (Affinity, 1:1000), Wnt3a (Affinity, 1:1000), GSK-3β (Affinity, 1:1000), p-GSK-3β (Affinity, 1:1000), Caspase-3 (Affinity, 1:1000), Caspase-9 (Affinity, 1:1000), Cyclin D1 (Proteintech, 1:1000), Survivin (Proteintech, 1:1000), Bcl-2 (Affinity, 1:1000), Bax (Affinity, 1:1000), GAPDH (Affinity, 1:5000), β-actin (Affinity, 1:5000), and IgG-HRP (Proteintech, 1:10000).

### Statistical analysis

The significance of mean differences between multiple groups was analyzed using one-way analysis of variance (ANOVA), and the student’s *t*-test was used for two groups [25]. *p* < 0.05 was set to be statistically significant. All assays were repeated three times. Iamge J software was used to process the images of cell wound healing, clones, Transwell and western blotting. The images of flow cytometry were processed by FlowJo software. And the data were analyzed by Prsim software.

## Results

### Erianin inhibited the proliferation of DDP-resistance NSCLC in vitro

**Fig. 1:**
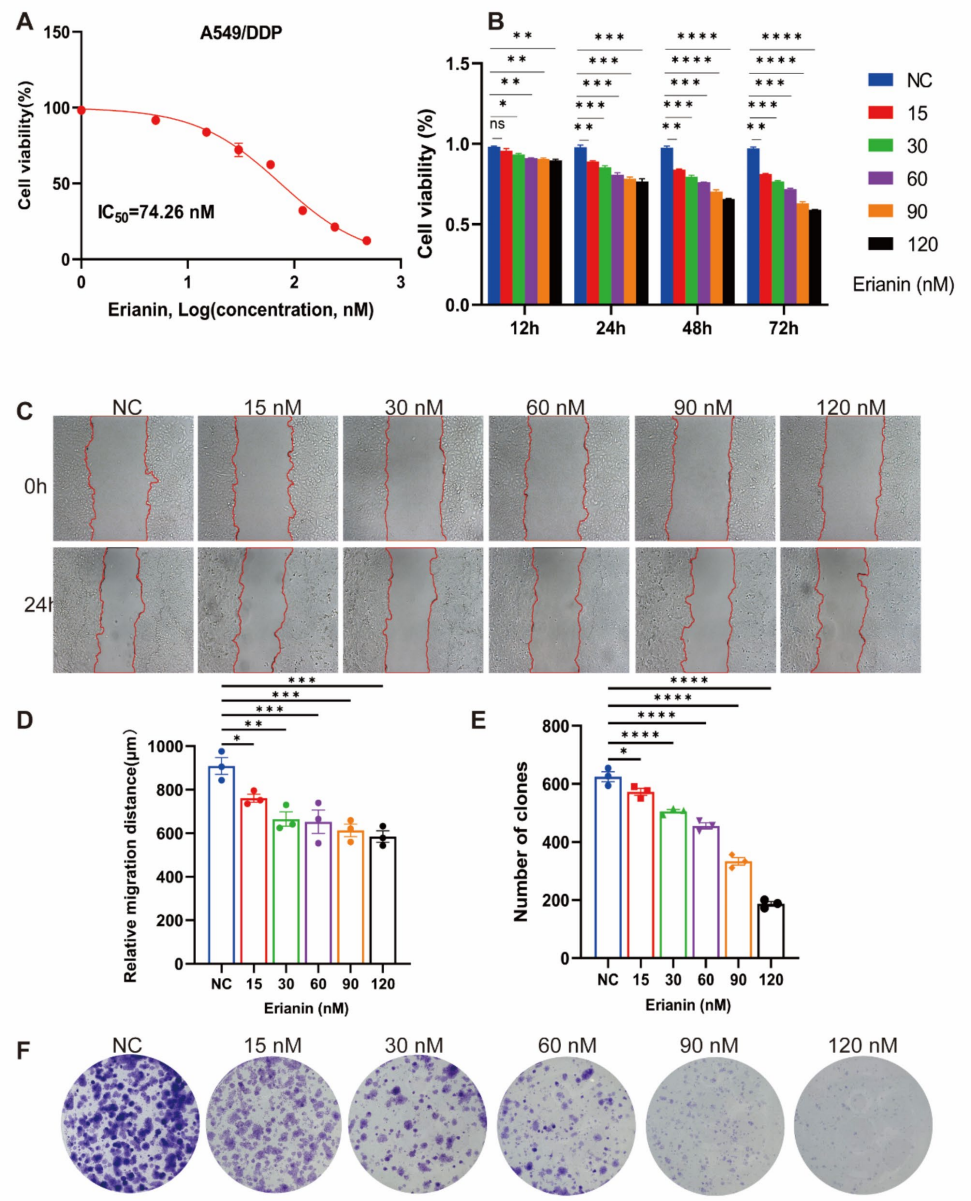
Effect of Erianin on proliferation of DDP-resistant LUAD cells in vitro. (**A**) The IC_50_ value of Eranin in A549/DDP cells was 74.26 nM. (**B**) The cell viability of A549/DDP cells after Eranin treatment was measured by CCK-8. The proliferation of A549/DDP cells after Eranin treatment was determined by (**C**–**D**) wound healing assay and (E-F) cell colony-forming assay. (ns: *p* > 0.05, *p < 0.05, **p < 0.01, ***p < 0.001, **** *p* < 0.0001)

We used the CCK-8 assay to detect the IC_50_ value. Results shown that the IC_50_ of Erianin was 74.26nM in DDP/A549 cells (Fig. 1A). Then, we established the concentration gradient based on the IC_50_ for subsequent assays. Next, we used CCK-8, wound healing and colony formation assay to detected the proliferation ability of cells. The results of CCK-8 showed that Erianin significantly inhibited the viability of A549/DDP cells (Fig. 1B), the higher the concentration of Erianin and the longer the action time, the more obvious the inhibitory effect on cell viability. In addition, after 24 h of treatment with Erianin, the wound healing of cells in the Erianin group was significantly slower than that in the control group (Fig. 1C-D). Similarly, the Erianin group also inhibited the ability of cells to clone into clusters (Fig. 1E-F), and the inhibitory effect became more significant with increasing Erianin concentration.

### Erianin inhibited the migration and invasion of DDP-resistance NSCLC in vitro

**Fig. 2:**
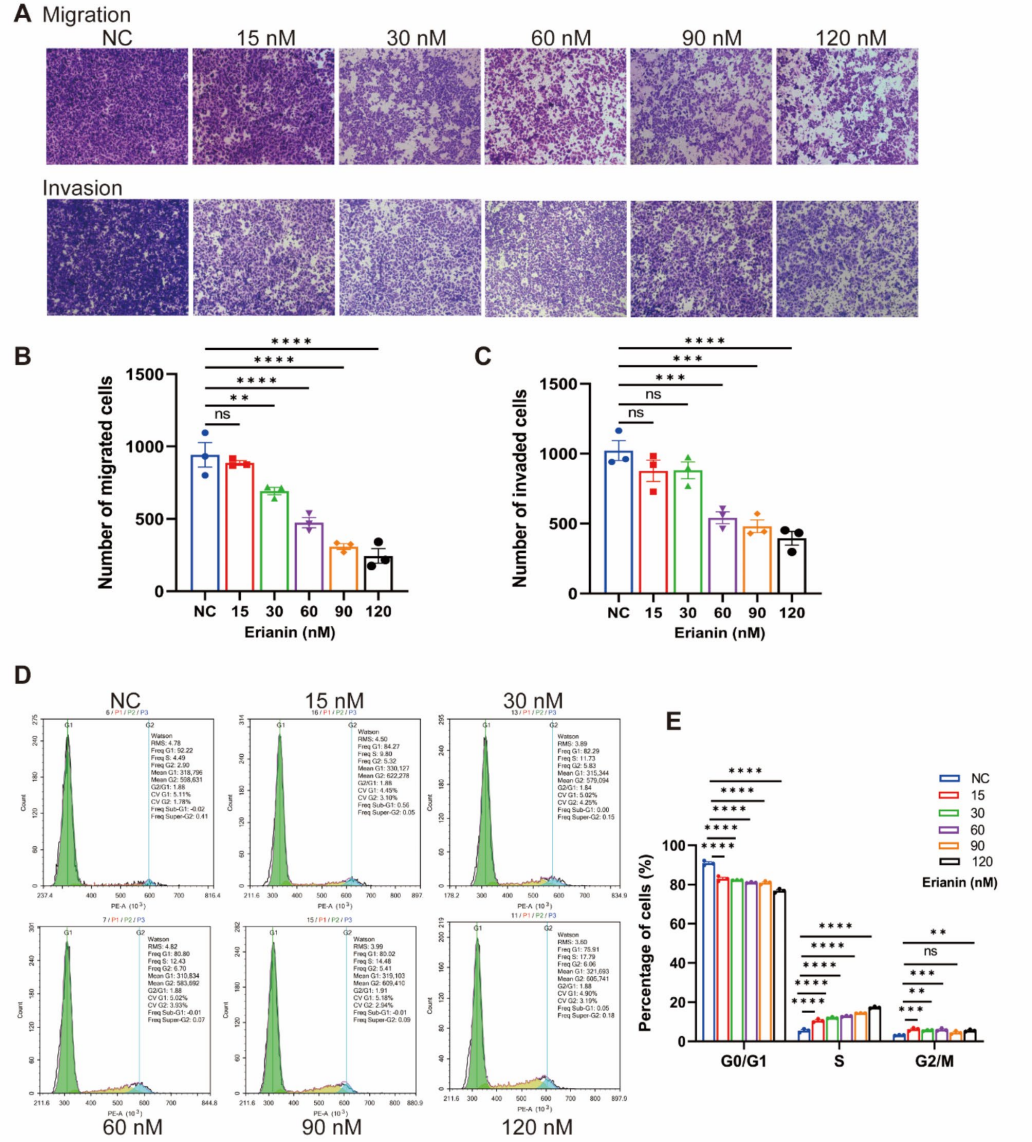
Effect of Erianin on migration and invasion of DDP-resistant LUAD cells in vitro. (**A**–**C**) The migration and invasion of A549/DDP cells after Eranin treatment were determined by Transwell assay. (**D**–**E**) And the cell cycle of A549/DDP cells after Eranin treatment was detected by flow cytometry. (ns: *p* > 0.05, *p < 0.05, **p < 0.01, ***p < 0.001, ****p < 0.0001)

We evaluated the effects of Erianin on migration and invasion of cells by Transwell assay. We found that the migration and invasion ability of A549/DDP cells after Erianin treatment was inhibited compared to the control group (Fig. 2A). In the range of 30 ~ 120 nM, the inhibition of Erianin on cell migration was statistically different (Fig. 2B). There were also statistical differences in cell invasion inhibition in the range of 60 ~ 120 nM (Fig. 2C).

### Effect of Erianin on cell cycle of DDP-resistance NSCLC in vitro

We detected the changes of cell cycle after treatment with Erianin by flow cytometry. Results showed that compared with the control group, the G0/G1 phase of the Erianin group was significantly shortened, the S and G2/M phases were significantly prolonged (Fig. 2D-E). Combined with the inhibitory effects of Erianin on proliferation detected by CCK-8, cell wound healing and colony assay, we hypothesized that Erianin plays its antitumor role by blocking the cell cycle of A549/DDP in S and G2/M phases.

### Erianin induced apoptosis of DDP-resistance NSCLC in vitro

**Fig. 3:**
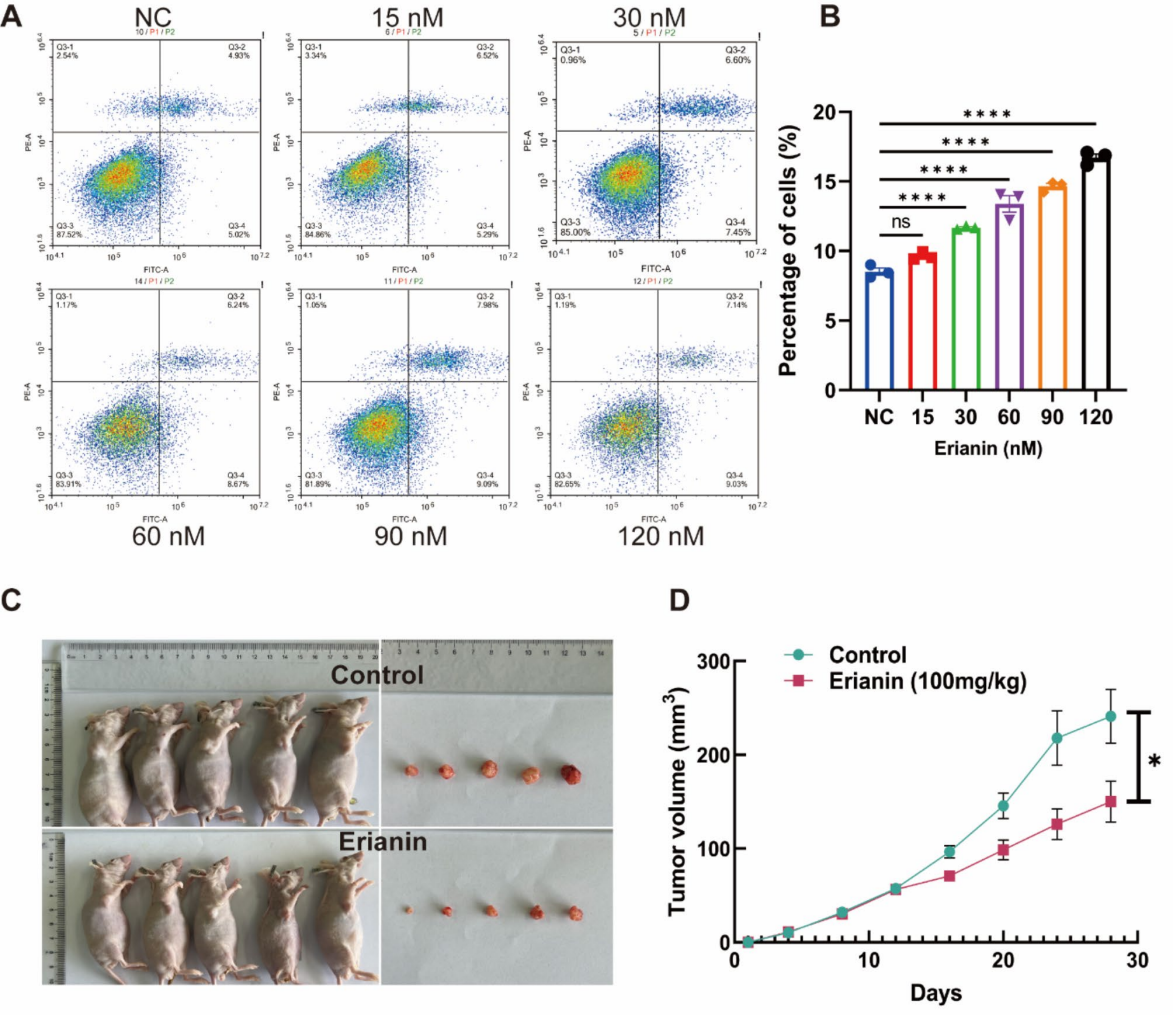
Effect of Erianin on apoptosis of DDP-resistant LUAD cells in vitro and in vivo. (**A**–**B**) The apoptosis of A549/DDP cells in vitro after Eranin treatment was determined by flow cytometry. (**C**–**D**) Tumor transplantation experiment in nude mice and growth curve of tumor tissue. (ns: *p* > 0.05, *p < 0.05, **p < 0.01, ***p < 0.001, **** *p* < 0.0001)

We detected the cell apoptosis by flow cytometry. Results showed that the apoptosis of A549/DDP cells after Erianin treatment was increased in vitro, and there were statistical differences in the concentration range of 30 ~ 129 nM (Fig. 3A-B).

### Erianin inhibited the proliferation and induced apoptosis of DDP-resistance NSCLC in vivo

In the transplantation tumor experiment, tumor growth of the control group and Erianin group were shown in Fig. 3C. During continuous administration, we found that the growth rate of transplanted tumor volume in Erianin group was slower than control group (Fig. 3D). And after 15 days of continuous treatment, the volume of transplanted tumors in Erianin group was also significantly decreased compared to the control group (Fig. 3D).

### Erianin reversed the resistance of DDP-resistant NSCLC to DDP

**Fig. 4:**
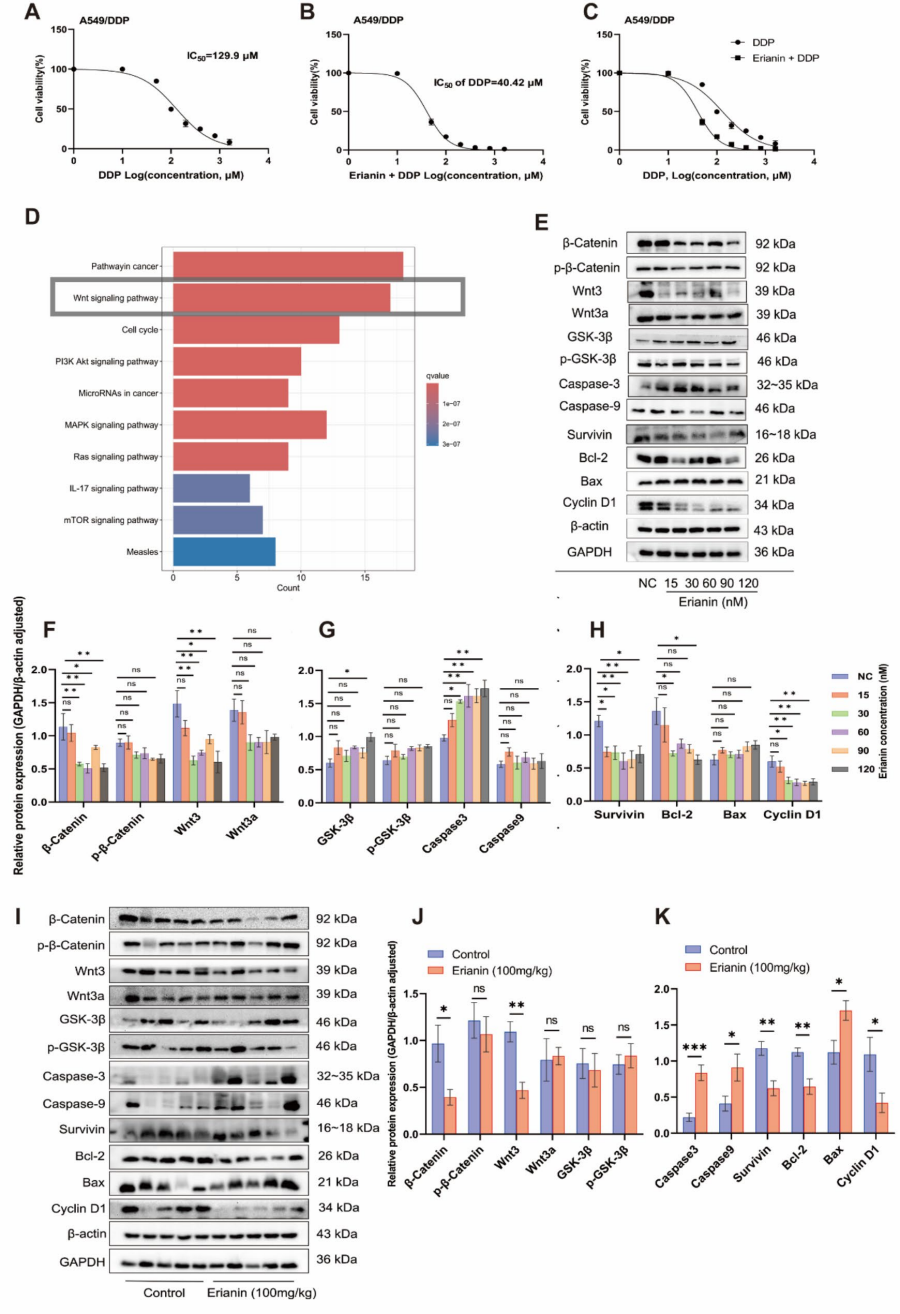
Effects of Erianin on drug resistance and protein expression of DDP-LUAD resistant cells in vivo and in vitro. (**A**–**C**) IC_50_ values of each group were detected by CCK-8. (**D**) Enrichment analysis of differentially expressed genes in cell models of Erianin group and control group. The proteins expression in the (**E**–**H**) in vitro cells models and (**I**–**K**) in vivo transplanted tumor tissues of the Erianin group and control group were detected by Western blotting. (ns: *p* > 0.05, *p < 0.05, **p < 0.01, ***p < 0.001, **** *p* < 0.0001)

We added DDP with different concentration (1 ~ 1000 µM) into the culture medium for the treatment of A549/DDP cells, and detected the cell viability by CCK-8 after 24 h. The results indicated that as the concentration of DDP increased, the viability of A549/DDP cells decreased. (Fig. 4A). Subsequently, A549/DDP cells were treated with Erianin in 74.26 nM (IC_50_) for 24 h, and then replaced with DDP at the same concentration as before (1 ~ 1000 µM) to treat A549/DDP cells. After 24 h, cell viability was detected again by CCK-8 assay. We found that at the same concentration of DDP, the combination therapy of Erianin and DDP had a higher inhibition rate on A549/DDP cell viability than DDP monotherapy (Fig. 4B-C). In addition, the IC50 value of DDP in the combination group was 40.42 µM, which was 3.21 times higher than the 129.9 µM value in the DDP monotherapy group.

### Erianin affected protein expression of DDP-resistant NSCLC in vitro and in vivo

In order to further explore the mechanism of action of Erianin on DDP-resistant LUAD, we constructed the cell models of Erianin treatment group and control group. Then, we predicted the potential molecular mechanisms by using RNA-seq technology (BMKCloud) and enrichment analysis. Results indicated that the differentially expressed genes between the two groups were significantly enriched in the “Wnt signaling pathway” (Fig. 4D). In addition, in the prediction of network pharmacological targets on the anti-tumor activity of Erianin, researchers also found that Erianin plays a positive regulatory role in non-small cell lung cancer, cell cycle, apoptosis, and cell migration [21]. Therefore, this study further examined the expression of related proteins in vitro and in vivo.

The western blotting results of cells showed (Fig. 4E-H) that after Erianin treatment, the expression levels of β-catenin, p-β-catenin, Wnt3, and Wnt3a proteins in A549/DDP cells were down-regulated compared to the control group, but only the down-regulation of β-catenin and Wnt3 proteins had statistical significance. GSK-3β and p-GSK-3β proteins were not significantly down-regulated in this study. In the detection of apoptosis and proliferation-related proteins, we found that Erianin can up-regulate the expression of Caspase3, Caspase9, and Bax proteins, and down-regulate the expression of Bcl-2 and Survivin proteins. Simultaneously significantly down-regulate the expression of Cyclin D1 protein. Among those proteins, only Survivin, Bcl-2, Caspase3, Cyclin D1 were statistically significant.

In the western blotting results of tissues (Fig. 4I-K), we found that Erianin significantly down-regulated the expression of β-catenin, Wnt3, Cyclin D1, Bcl-2, and Survivin proteins, and significantly up-regulated the expression of Caspase3 protein, which is consistent with the results of cell models. Different from cell models, the expression of Bax protein in tumor tissue was also significantly increased after the treatment of Erianin, which may be due to the higher dose of drugs used in vivo and the more obvious therapeutic effect.

## Discussion

As a broad-spectrum anticancer drug, Erianin has significant inhibitory effect on multiple tumor types through various mechanisms in vivo and in vitro. Tumor growth is closely related to the imbalance between cell proliferation and death, and the apoptosis of tumor cells has a negative effect on tumor and can inhibit their rapid growth. Inducing apoptosis, inhibiting migration, invasion, and proliferation are the key pathways and ultimate goals of most anticancer treatments, including Erianin [36, 37].

In terms of cell proliferation, the inhibitory effect of Erianin on many types of cancers has been reported. For instance, Erianin inhibited the pancreatic cancer cell proliferation through AKT/FOXO1 and ASK1/JNK/p38 MAPK signaling pathways [38]. Erianin has obvious inhibitory effect on the growth of hepatocellular transplanted tumor mice, but has no obvious toxic effect on mice [39]. Eranin also significantly inhibited the cell proliferation in esophageal squamous cell carcinoma and triple negative breast cancer cell lines, and inhibited the tumor growth in transplanted tumor models [40, 41]. In terms of the cell cycle, Cyclin D1 is an important regulator in the G0/G1 phase [42], and its expression in normal cells is tightly regulated, but its activity is abnormally enhanced in various ways in some cancers [43]. High expression of Cyclin D1 drives uninhibited cell proliferation and promotes tumor growth, thus, Cyclin D1 plays a central role in the pathogenesis of cancer [44]. According to the prediction of Yan et al., Cyclin D1 was also a target of Erianin in the treatment of lung cancer, cervical cancer, bladder cancer, colorectal cancer and breast cancer [21]. In this study, we observed that Erianin could inhibit the proliferation of DDP-resistant LUAD cells. And, we also confirmed that Erianin can block the cell cycle of DDP-resistant LUAD cells in the S and G2/M phases, and down-regulate the expression of Cyclin D1 protein to exert its anti-tumor effects.

In terms of apoptosis, Erianin is also involved in the programmed death of many types of cancer cells. The activation of caspase cascade reactions after Erianin treatment was significantly increased, and the changes of mitochondrial membrane potential were directly related to the apoptosis pathway of nasopharyngeal carcinoma cells [21]. Erianin also up-regulated the expression of caspase-3, -7, -9 in breast cancer cells, and altered the ratio of Bcl-2/Bax to induce cell apoptosis [45]. In cervical cancer cells, Erianin induced mitochondrial apoptosis by regulating the expression of ERK and p53, and play a regulatory role in the expression of Bcl-2 and Bax [46]. In the study of Han et al., they also found that Erianin can induce apoptosis of cancer cells by activating Caspase3 and Bax in NSCLC cells via the Akt-GSK3β signaling pathway [47]. In this study, we observed that Erianin induced cell apoptosis, and significantly up-regulated the apoptosis related protein Caspase3 and down-regulate the anti-apoptosis related proteins Bcl-2 and Survivin in DDP-resistance LUAD. Apoptosis is a mode of programmed cell death, Erianin can promote the apoptosis of tumor cells by up-regulating the expression of Caspase 3 [29, 38]. This mechanism of action helps to eliminate abnormal cells in the body and maintain normal physiological functions of the body. Bcl-2 is an apoptosis suppressor gene, and its protein product has the effect of prolonging cell survival [48, 49]. Under normal circumstances, Bcl-2 can maintain the normal physiological function of cells and prevent excessive cell apoptosis. However, in tumor cells, high expression of Bcl-2 is often associated with increased anti-apoptotic ability and increased drug resistance of tumor cells [50, 51]. By down-regulating the expression of Bcl-2, Erianin can weaken the anti-apoptotic ability of tumor cells, making them more susceptible to induction of apoptosis signals. This mechanism helps to enhance the sensitivity of tumor cells to chemotherapy drugs and improve the therapeutic effect [52, 53]. Furthermore, in the evaluation of drug resistance, we found that A549/DDP cells incubated with Erianin for 24 h were more sensitive to DDP treatment. We hypothesized that Erianin treatment could improve the sensitivity of DDP-resistant cells to chemotherapy, and this mechanism may be accomplished by down-regulating Bcl-2.

Survivin, a new member of the apoptosis suppressor protein family, is tumor-specific and expressed only in tumors and embryonic tissues [54]. High expression of Survivin is closely related to the differentiation, proliferation, invasion and metastasis of tumor cells [55]. We found that Erianin inhibited the proliferation, invasion and metastasis of tumor cells by down-regulating Survivin expression. This mechanism of action helps to control the growth and spread of tumors and improve the survival rate of patients. Therefore, Survivin is also a valuable therapeutic target for DDP-resistance LUAD.

In addition, our study found that Erianin significantly activated the expression of Caspase9 in transplanted tumor, but did not significantly activate in cell lines, which may involve a variety of complex factors. In the nude mouse transplanted tumor model, the tumor cells were placed in a microenvironment that was completely different from in vitro cell line culture. This environment includes extracellular matrix, blood vessels, immune cells, and other stromal cells that may interact with tumor cells to affect Caspase9 activation. In contrast, cultured cell lines may lack the stimulation of these growth factors and signaling molecules. In nude mice, Erianin may be metabolized by liver, kidney and other organs to produce metabolites with different biological activities. These metabolites may have a stronger ability to activate Caspase9. However, in vitro cell lines, Erianin acts directly on tumor cells without metabolism, which may not effectively activate Caspase9.

Previous study has confirmed that Erianin can regulate the pyruvate carboxylase-mediated Wnt/β-Catenin pathway in human hepatoma [56]. In this study, enrichment analysis based on RNA-seq also detected significant enrichment of differentially expressed genes in the Wnt pathway between the Erianin treatment group and the control group. The Wnt/β-catenin pathway, also known as the typical Wnt signaling pathway, is a conserved key axis that coordinates multiple cellular signaling cascades [57, 58]. It plays an indispensable role in physiological processes including cell proliferation, differentiation, apoptosis, migration, invasion, and tissue homeostasis. This pathway is involved in the carcinogenesis process of various cancers and is one of the potential cancer treatment targets [59]. Some pathway inhibitors in preclinical research, such as curcumin and vitamin D, have been identified to inhibit tumor progression [60–62]. Therefore, there is still great potential for the development of effective drugs targeting the Wnt pathway. β-catenin is an important component and down-stream effector of the Wnt signaling cascade [63]. GSK-3β is a serine/threonine kinase that phosphorylates β-catenin residues Ser33, Ser37, and Thr41 [64]. These phosphorylation events drive the ubiquitination and proteasomal degradation of beta-catenin, resulting in only small amounts of β-catenin being maintained in unstimulated cells [65]. Dysregulation of the Wnt pathway is often involved in the pathogenesis of serious diseases including liver cancer, lung cancer. breast cancer, stomach and prostate cancer [66, 67]. Cyclin D1 is also one of its target genes. In addition, abnormal activation of the Wnt pathway has been shown to promote the proliferation, renewal, and differentiation of cancer stem cells, leading to the development of chemotherapy resistance and immune evasion [68].

Abnormal activation of the Wnt/β-Catenin pathway is also involved in multi-drug resistance in various cancers, including DDP-resistance in ovarian and prostate cancers. In this study, we observed that Erianin reversed the resistance to DDP, and down-regulated the expression of Wnt3 and β-Catenin proteins in DDP-resistant LUAD cells. Previous research has reported that Erianin also can down-regulated the expression of p-GSK-3β protein, and play a benign role in precancerous lesions of gastric cancer by inhibiting the HRAS-PI3K-AKT signaling pathway [69]. However, in our study, significant down-regulation of GSK-3β or p-GSK-3β was not been confirmed.

In summary, this study suggested that Erianin has the potential to induce cell apoptosis, inhibit cell proliferation and migration, altered cell cycle in DDP-resistance LUAD cells, which may be accomplished through the Wnt3/β-Catenin/Survivin/Caspase3/Bcl-2/Cyclin D1 axis. And the conclusions provide theoretical support for Erianin as a potential compound for the treatment of DDP-resistance LUAD.

## Conclusion

Erianin may exert its anti-tumor effect in DDP-resistance LUAD cells by regulating the Wnt3/β-Catenin/Survivin/Bcl-2/Caspase3/Cyclin D1 axis.

## Data Availability

All data generated or analyzed during this study are included in the manuscript.
